# Editorial: Contribution of artificial intelligence-based tools to the study of Parkinson's disease and other movement disorders

**DOI:** 10.3389/fnagi.2025.1567706

**Published:** 2025-03-04

**Authors:** Matilde Otero-Losada, Santiago Perez Lloret, Francisco Capani, Cristian Falup-Pecurariu

**Affiliations:** ^1^Centro de Altos Estudios en Ciencias Humanas y de la Salud, Universidad Abierta Interamericana, Consejo Nacional de Investigaciones Científicas y Técnicas, CAECIHS.UAI-CONICET, Buenos Aires, Argentina; ^2^Instituto Universitario de Ciencias de la Salud, Fundación H.A. Barceló, Consejo Nacional de Investigaciones Científicas y Técnicas (CONICET), Buenos Aires, Argentina; ^3^Departamento de Fisiología, Facultad de Medicina, Universidad de Buenos Aires (UBA), Buenos Aires, Argentina; ^4^Instituto de Ciencias Biomédicas, Facultad de Ciencias de la Salud, Universidad Autónoma de Chile, Santiago, Chile; ^5^Department of Neurology, Transilvania University of Brasov, Brasov, Romania

**Keywords:** artificial intelligence, machine learning, Parkinson, dopamine, autism

Parkinson's disease is the second most frequent neurodegenerative disorder, affecting about 1% of adults over 60 worldwide. Other movement disorders like Multiple System Atrophy, Huntington's Disease, Dystonia, or cerebellar ataxias may be less common but severely impair patients' life quality. Not only is the pathophysiology of many of these diseases incompletely understood, but diagnostic tools and therapeutic interventions are often insufficient as well. Machine learning (ML) is a central feature of Artificial Intelligence (AI)—the computer-based intelligence, able to perform typically human-like tasks. The applications of AI and ML within healthcare settings might be engaged in developing and applying new methods for disease diagnosis and treatment, drug discovery processes, and delving into the pathophysiology of some conditions.

Here, we present a few scientific articles using AI/ML-based tools in diagnosis, prognosis, and treatment of Parkinson's disease and other movement disorders, including others also characterized by dopaminergic dysfunction. These are:

*Classification of Parkinson's disease by deep learning on midbrain MRI*. The authors compared diagnostic performance of four approaches in PD patients and healthy controls (Welton et al.). Susceptibility map weighted imaging (SMWI), based on quantitative susceptibility mapping (QSM), allows accurate nigrosome-1 (N1) evaluation, and has been used to develop Parkinson's disease (PD) deep learning (DL) classification algorithms. Neuromelanin-sensitive (NMS) MRI could improve automated quantitative N1 analysis by revealing neuromelanin content (Fu et al., 2016; Shin et al., 2021; Sung et al., 2019). The four diagnostic approaches compared in this study were: (1) N1 quantitative “QSM-NMS” composite marker, (2) DL model for N1 morphological abnormality using SMWI (“Heuron IPD”), (3) DL model for N1 volume using SMWI (“Heuron NI”), and (4) N1 SMWI neuroradiological evaluation. The data demonstrated excellent performance of a quantitative QSM-NMS marker and automated DL PD classification algorithms based on midbrain MRI, while suggesting potential further improvements. Clinical utility is supported but requires validation in earlier stage PD cohorts.

*Detection of freezing of gait in Parkinson's disease from foot-pressure sensing insoles using a temporal convolutional neural network*. Freezing of gait (FoG) is a common and debilitating symptom of PD that can lead to falls and reduced quality of life (Park et al.). Wearable sensors have been used to detect FoG, but current methods have limitations in accuracy and practicality (San-Segundo et al., 2019; Shalin et al., 2021; Pardoel et al., 2019). This study aimed to develop a deep learning model using pressure sensor data from wearable insoles to accurately detect FoG in PD patients. A temporal convolutional neural network (TCNN) model was proposed. It achieved the highest accuracy, precision, sensitivity, specificity, and F1 score for FoG detection compared to other models and showed good performance in detecting FoG episodes, proving the potential of using wearable pressure sensors and machine learning models for FoG detection in PD patients. The TCNN model might be used to develop a real-time FoG detection system to improve PD patients' safety and quality of life.

*Machine learning model comparison for freezing of gait prediction in advanced Parkinson's disease*. The prevalence of the paroxysmal motor phenomenon known as Freezing of gait (FOG) increases as PD progresses (Watts et al.). Precision-based detection and classification of freezers are critical to developing tailored treatments rooted in kinematic assessments. This study analyzed instrumented stand-and-walk (SAW) trials from advanced PD patients with sub-thalamic nucleus deep brain stimulation (STN-DBS). A total of 21 PD subjects were evaluated (average age 64.24 years, 16 males, mean disease duration of 14 years). Analysis of random forests' feature estimation revealed the top-ten spatiotemporal predictive features utilized in the model: foot strike angle, coronal range of motion [trunk and lumbar], stride length, gait speed, lateral step variability, and toe-off angle. The results showed that machine learning effectively classified advanced PD patients as freezers or non-freezers based on SAW trials in their non-medicated/non-stimulated condition (Mancini et al., 2023; O'Day et al., 2020; Ramdhani et al., 2023).

*An automated hybrid approach via deep learning and radiomics focused on the midbrain and substantia nigra to detect early-stage Parkinson's disease*. The altered neuromelanin in substantia nigra pars compacta (SNpc) is a valuable biomarker in the detection of early-stage PD (EPD) (Chen et al.). Diagnosis via visual inspection or single radiomics based method is challenging. The authors proposed a novel hybrid model that integrates radiomics and deep learning methodologies to automatically detect EPD based on neuromelanin-sensitive MRI, namely short-echo-time Magnitude (setMag) reconstructed from quantitative susceptibility mapping (QSM). They collected QSM images including 73 EPD patients and 65 healthy controls. Classified models based on radiomics features, deep learning features, and the hybrid of both were established through machine learning algorithms, respectively. The results showed that integrating deep learning and radiomic features presented a potent strategy for EPD computer-aided diagnosis (Gaurav et al., 2021; Huddleston et al., 2018; Le Berre et al., 2019).

*Using sustained vowels to identify patients with mild Parkinson's disease in a Chinese dataset*. Accurate PD diagnosis and treatment in the early stages can slow down disease progression (Wang et al.). However, this is challenging even for movement disorder specialists when modified Hoehn-Yahr staging (mH&Y) < 1.8. Dysarthria provides good indicators for computer-assisted diagnosis of patients with PD. Few studies have focused on diagnosing PD patients in the early stages, specifically those with mH&Y ≤ 1.5. A machine learning algorithm analyzed voice features and developed diagnostic models for differentiating between healthy controls (HCs) and PD patients or patients with mild PD (mH&Y ≤ 1.5). The results showed a remarkable diagnostic performance of the model in differentiating patients with mild PD (mH&Y ≤ 1.5) and HCs, with area under the ROC curve 0.93 (95% CI: 0.85–1.00), accuracy 0.85, sensitivity 0.95, and specificity 0.75. This approach may be relevant in primary medical institutions with little availability of movement disorder specialists and special equipment shortage (Postuma et al., 2012; Song et al., 2020; Rusz et al., 2021).

*Enhancing attention in autism spectrum disorder: comparative analysis of virtual reality-based training programs using physiological data*. Attention deficit disorder (ADD) is a common symptom associated with autism spectrum disorder (ASD), challenging and impacting social interactions and learning abilities (Sanku et al.). Virtual reality (VR) has emerged as a promising tool for attention training with the ability to create personalized virtual worlds, providing a conducive platform for attention-focused interventions. This was a preliminary study to develop a functional prototype for attention therapy systems. The aim at this stage was to create a framework called VR-PDA (Virtual Reality Physiological Data Analysis) that utilizes physiological data for tracking and improving attention in individuals. The results revealed that reinforcement training strategies are crucial for improving attention in ASD individuals. Among different strategies, the noise strategy had superior efficacy in training attention in ASD individuals. No specific training proved effective in enhancing attention in non-ASD individuals. These findings provide valuable insights into the effectiveness of different strategies for attention training and emphasize the potential of virtual reality (VR) and physiological data in attention training programs for individuals with ASD (Hodges et al., 2020; Hellum et al., 2023; Patel et al., 2023).

The findings from the studies included in this Research Topic are inherently valuable, paving the way for future research and innovations.
